# Association of corticosteroids and biologics use for inflammatory bowel disease with surgical outcomes following knee or hip arthroplasty

**DOI:** 10.1093/crocol/otag119

**Published:** 2026-11-12

**Authors:** Passisd Laoveeravat, Tarek Odah, Hassan Ghoz, Jana G Hashash, Francis A Farraye

**Affiliations:** Division of Gastroenterology and Hepatology, Mayo Clinic, Jacksonville, Florida, United States; Division of Gastroenterology and Hepatology, Mayo Clinic, Jacksonville, Florida, United States; Division of Gastroenterology and Hepatology, Mayo Clinic, Jacksonville, Florida, United States; Division of Gastroenterology and Hepatology, Mayo Clinic, Jacksonville, Florida, United States; Division of Gastroenterology and Hepatology, Mayo Clinic, Jacksonville, Florida, United States

**Keywords:** anti-tumor necrosis factor agents, arthroplasty, corticosteroids, inflammatory bowel disease, orthopedic outcomes

## Abstract

**Background:**

The impact of corticosteroid and biologic therapy use among patients with inflammatory bowel disease (IBD) on postoperative complications in arthroplasties remains uncertain. This study aims to evaluate the association between preoperative corticosteroids or anti-tumor necrosis factor (TNF) use in patients with IBD and the risk of orthopedic-specific complications following arthroplasty.

**Methods:**

This retrospective study utilized TriNetX with propensity score matching. Patients prescribed corticosteroids or anti-TNF medications, within 3 months prior to hip or knee arthroplasty were included. Outcomes included prosthetic joint infection (PJI), prosthesis dislocation, and wound disruption categorized into early and late events.

**Results:**

In corticosteroid cohort, early PJI and prosthesis dislocation risk were higher compared to controls with odds ratio (OR) of 1.71 (95% confidence interval [CI] 1.08-2.72; *P* = .021) and 2.03 (95% CI 1.17-3.53; *P* = .010), respectively. Wound disruption was not different between groups. In patients on anti-TNFs, the risk of early and late PJI were not significantly increased in patients compared to controls, ORs of 1.08 (95% CI 0.65-1.80; *P* = .76) and 1.79 (95% CI 0.88-3.64; *P* = .11), respectively. Early prosthesis dislocation and wound disruption risks were not increased.

**Conclusions:**

Preoperative corticosteroid use in patients with IBD was associated with increased early risks of PJI and dislocation after arthroplasty. Preoperative anti-TNF exposure was not associated with a statistically significant increase in adverse orthopedic outcomes. These findings highlight the importance of minimizing perioperative corticosteroids while suggesting that recent anti-TNF exposure might not be associated with increased surgical complications. Further research is needed to confirm these findings.

## Teaser

Summary

Corticosteroid use increased the risk of infection and joint dislocation in patients with inflammatory bowel disease undergoing hip or knee replacement surgery. However, anti-tumor necrosis factors are safe for use prior to joint surgery.

Key Messages **What is already known?** 

- Corticosteroids are associated with increased risk of any infection post arthroplasty but has not been explored in the orthopedic specific outcomes.
- Anti-tumor necrosis factors have been recommended to be held prior to the arthroplasty without clear evidence.

**What is new here?** 

- Corticosteroid use was associated with higher prosthetic joint infection and joint dislocation after arthroplasty.
- Anti-tumor necrosis factor therapy within 3 months prior to arthroplasty was not associated with a statistically significant increase in adverse orthopedic outcomes.

**How can this study help patient care?** 

- This study will help guide the perioperative management of corticosteroids and anti-TNF agents in patients with inflammatory bowel disease undergoing arthroplasty surgery to reduce the postoperative complications and inflammatory bowel disease flare.

## Introduction

Inflammatory bowel disease (IBD) including ulcerative colitis (UC) and Crohn’s disease (CD) is a chronic, relapsing and remitting disorder associated with gastrointestinal tract inflammation and systemic immune dysregulation.^1^^,^^2^ Patients with IBD may require orthopedic interventions such as total hip arthroplasty (THA) and total knee arthroplasty (TKA). The perioperative management of patients with IBD undergoing arthroplasty presents unique challenges. Orthopedic surgeons may require that patients be off corticosteroids, infliximab and other IBD medications prior to joint surgery for several weeks prior to surgery and to remain off of these medications for several weeks post-surgery, potentially leading to a prolonged interruption of therapy. Stopping anti-tumor necrosis factor (anti-TNF) agents such as infliximab or adalimumab for a prolonged period of time puts patients at risk for flare up of their IBD and increased risk of immunogenicity as restarting may lead to development of anti-drug antibodies or infusion reactions precluding continued use.^3^^,^^4^

Prior studies demonstrated that patients with IBD have increased rates of postoperative complications,^5–7^ including periprosthetic joint infection (PJI), non-joint related infections (pneumonia or *Clostridoides difficile* infection) and prosthesis dislocation. Registry based studies indicate that patients with IBD have a higher risk of revision surgery for septic causes following THA. One reported a 5- and 4-fold increased risk of PJI and joint revisions, respectively.^5^

The impact of IBD related therapies on arthroplasty outcomes varies among different therapies. Corticosteroid (>10 mg/d) and infliximab use are associated with 3.8% and 2.5% risk of postoperative joint infection, respectively, in patients with rheumatoid arthritis.^8^ Meta-analysis on anti-TNFs contributes to 1.5 times of post-operative overall infection complications.^9^ There was only 1 study on infliximab and PJI in patients with any rheumatological disorder.^10^ No studies have been conducted to include patients with IBD on therapies and post arthroplasty orthopedic outcomes.

There is no definite guideline informing clinical practice for patients with IBD on biologic therapies undergoing hip or knee arthroplasty. However, the guidelines in patients with rheumatic diseases undergoing joint replacement surgery recommended withholding 1 dose of all biologics prior to the surgery and planning that the surgery take place after skipped dose to aim for lowest drug level at the surgery time.^11^

We aimed to evaluate the association between preoperative corticosteroid or anti-TNF use in patients with IBD and the risk of PJI, prosthesis dislocation, and wound disruption following arthroplasty.

## Materials and methods

### Data source

A retrospective cohort study was conducted utilizing the U.S. collaborative network in TriNetX, a federated health research network that integrates de-identified electronic health records with medical and pharmacy claim data from various healthcare organizations. The Linked Network provides comprehensive, longitudinal data on patient journeys, allowing for robust analyses of demographics, clinical encounters, medication usage, laboratory results, and outcomes.

### Study participants

All study participants were adults (≥18 years) who underwent hip or knee surgery (initial or revision), as defined by the procedure codes listed in Table S1.

## Medication exposure and comparisons

Within this surgical population, IBD-directed medications were defined as TNF inhibitors (infliximab, adalimumab, certolizumab pegol, golimumab) and non-TNF inhibitor advanced therapies (vedolizumab, natalizumab, ustekinumab, risankizumab, guselkumab, mirikizumab, upadacitinib, tofacitinib, ozanimod, and etrasimod). Two comparisons were examined: (1) patients with IBD (CD [K50] or UC [K51]) receiving oral or intravenous steroids (methylprednisolone, dexamethasone, hydrocortisone, prednisolone, or prednisone), which were pooled together regardless of dose or duration, but not IBD-directed medications versus those not receiving steroids or IBD-directed medications; (2) patients receiving TNF inhibitors (infliximab, adalimumab, certolizumab pegol, or golimumab but not non-TNF inhibitor advanced therapies) versus those not receiving any IBD-directed medications. Medication exposure was defined as prescription for these medications within the 3 months preceding hip or knee surgery. The TriNetX database does not allow determination of medication dose, duration, adherence, precise timing relative to surgery, or whether therapy was held immediately before surgery. For the 2 comparisons, cohorts were propensity-matched on age, sex (female), White race/ethnicity, body mass index category (15-18.5, 18.5-25, 25-30, 30-35, 35-40, 40-50, 50-60, and 60-80 kg/m^2^), nicotine dependence, malnutrition, and diabetes; oral/IV steroid use was additionally included as a matching variable in the second comparison.

## Outcomes

Study outcomes included PJI, wound disruption, and prosthetic dislocation or instability of the hip or knee. Each outcome was evaluated separately for early (≤3 months) and late (3-12 months) postoperative periods. PJI was identified using ICD-10-CM codes T84.51, T84.52, T84.53, and T84.54; wound disruption was defined by code T81.3; and dislocation or instability of hip or knee prostheses was identified using codes T84.020, T84.021, T84.022, and T84.023, respectively.

### Statistical analysis

Analyses were conducted using the TriNetX research network platform. Propensity score matching (1:1 nearest-neighbor matching with a caliper of 0.1 pooled standard deviations) was used to balance covariates between cohorts. After matching, outcomes were compared using odds ratios (ORs) with 95% confidence intervals (CIs) and corresponding *P*-values. A 2-sided *P* < .05 was considered statistically significant.

## Results

### Cohort identification and matching

For the TNF inhibitor analysis, 2409 exposed patients and 853 002 controls were identified for the ≤3-month analysis, and 3125 exposed and 1 073  813 controls for the 3-12-month analysis. For the steroid analysis, 8992 steroid-treated patients and 4908 controls were identified for the ≤3-month cohort, and 8924 and 5076 patients receiving steroids and control, respectively, for the 3-12-month cohort.

After 1:1 propensity-score matching, 2338 patients per group were included in the TNF inhibitor analysis for outcomes occurring within ≤3 months, and 3078 patients per group for the 3-12-month analysis. In the steroid analysis, 4475 patients per group were included for the ≤3-month window and 4610 per group for the 3-12-month window. Covariate balance was achieved across all comparisons, with standardized mean differences below 0.1 for key variables.

Post-matching, the mean age was 61.8 ± 12 years in the anti-TNF cohorts and 66.6 ± 11 years in the steroid cohorts. Female patients represented 66% and 58% of each respective cohort, and 77%-79% were White. The mean body mass index was approximately 30-31 kg/m^2^. Comorbidities were similar across groups, with diabetes in 20%-25% of patients, nicotine dependence in 12%-14%, and malnutrition in 3%-4%. Median follow-up was 90 days for the early window and 365 days for the late window.

### Early postoperative outcomes (≤3 months)

In the TNF inhibitor analysis, PJI occurred in 1.36% of TNF users and 1.26% of controls (OR 1.08; 95% CI, 0.65-1.80; *P* = .76) as shown in Table 1. Wound disruption occurred in 1.53% and 1.65%, respectively (OR 0.93; 95% CI, 0.58-1.47; *P* = .75). Dislocation occurred in 0.83% of anti-TNF users and 0.61% of controls (OR 1.38; 95% CI, 0.69-2.75; *P* = .36).

**Table 1:** Impact of preoperative tumor necrosis factor (TNF) inhibitors or corticosteroids on arthroplasty outcomes.

Outcomes	Cohort	Exposed group (%)	Control (%)	Odds ratio (95% confidence interval [CI])	*P* value
**Early prosthetic joint infection (PJI) (≤3 months)**	TNF inhibitors^a^	1.36	1.26	1.08 (0.65-1.80)	.76
	Corticosteroids	1.12	0.66	1.71 (1.08-2.72)	.021
**Late PJI (3-12 months)**	TNF inhibitors^a^	0.71	0.40	1.79 (0.88-3.64)	.11
	Corticosteroids	0.43	< 0.22	Not reported^b^	–
**Early wound disruption (≤3 months)**	TNF inhibitors^a^	1.53	1.65	0.93 (0.58-1.47)	.75
	Corticosteroids	1.23	1.07	1.16 (0.78-1.72)	.47
**Late wound disruption (3-12 months)**	TNF inhibitors^a^	0.71	0.44	1.63 (0.82-3.26)	.16
	Corticosteroids	0.27	0.27	1.01 (0.45-2.24)	.99
**Early dislocation (≤3 months)**	TNF inhibitors^a^	0.83	0.61	1.38 (0.69-2.75)	.36
	Corticosteroids	0.87	0.43	2.03 (1.17-3.53)	.010
**Late dislocation (3-12 months)**	TNF inhibitors^a^	Not reported due to low event rates			
	Corticosteroids	Not reported due to low event rates			

a TNF inhibitors include infliximab, adalimumab, certolizumab, or golimumab.

b Not reported due to low event rate and patient privacy restrictions.

In the steroid analysis, PJI occurred in 1.12% of steroid-treated patients and 0.66% of controls, an absolute difference of 0.46% (OR 1.71; 95% CI, 1.08-2.72; *P* = .021). Wound disruption occurred in 1.23% and 1.07% of patients, respectively (OR 1.16; 95% CI, 0.78-1.72; *P* = .47). Dislocation occurred in 0.87% of steroid users and 0.43% of controls, an absolute difference of 0.44% (OR 2.03; 95% CI, 1.17-3.53; *P* = .010).

### Late postoperative outcomes (3-12 months)

Between 3 and 12 months after arthroplasty, PJI occurred in 0.71% of anti-TNF users and 0.40% of controls (OR 1.79; 95% CI, 0.88-3.64; *P* = .11). Wound disruption occurred in 0.71% and 0.44%, respectively (OR 1.63; 95% CI, 0.82-3.26; *P* = .16). Dislocation events were rare and not reported due to low event rate.

In the steroid cohort, PJI occurred in 0.43% of steroid-treated patients and less than 0.22% of controls; statistical estimates are not reported due to low event rate. Wound disruption occurred in 0.27% of both groups (OR 1.01; 95% CI, 0.45-2.24; *P* = .99). Dislocation events were rare and not reported due to low event rate.

## Discussion

Our study is among the first to evaluate the association of anti-TNF and corticosteroid exposure with orthopedic specific adverse outcomes post arthroplasty in patients with IBD. Corticosteroids use was associated with a higher risk of periprosthetic infection. Anti-TNFs were not associated with a statistically significant increase in risk of PJI. Additionally, immediate postoperative joint dislocation was associated with corticosteroids, not anti-TNFs use.

Our findings on corticosteroids and PJI are consistent with prior data on surgical site infection risk in patients with IBD undergoing joint surgery.^12^ Previous studies demonstrated that corticosteroid doses above 10 mg per day were associated with 2-fold increased risk of PJI risk compared to no corticosteroid use in patients with rheumatoid arthritis.^13^ Specifically, corticosteroid use in patients with IBD were associated with increased risk of any 90-day postoperative infection following joint replacement surgery with hazard ratio of 4.6. In contrast, anti**-**TNFs were not associated with a statistically significant increase in postoperative infection risk.^14^ Our study has shown no increased risk of wound disruption in patients who took corticosteroids and anti-TNFs prior to the hip and knee arthroplasty. One study showed a significant increase of 30-day wound dehiscence risk in patients taking corticosteroid prior to total joint arthroplasty.^15^

A prior study^10^ examined patients with any immune mediated disease including IBD, rheumatoid arthritis, ankylosing spondylitis, psoriatic arthritis on different timing of last dose of infliximab prior to the joint surgery (less than 4 weeks, 4-8 weeks, 8-12 weeks, 12-16 weeks, longer than 16 weeks prior to the surgery date). In that study, there was no increased risk of PJI in any timing of last infliximab administration. However, corticosteroids were associated with increased risk of PJI especially in corticosteroid doses higher than 10 mg per day. The study also investigated the timing of safe resumption of biologics after joint arthroplasty. Patients who restarted infliximab at any time had lower PJI rates than those who had not yet reinitiated it with hazard ratios of 0.50.^10^

Interestingly, corticosteroids exposure prior to hip or knee arthroplasty was associated with higher odds of postsurgical joint dislocation within 3 months postoperatively. Joint dislocation after knee and hip arthroplasty is associated with multiple risk factors including advanced age, female gender, obesity, neuromuscular disorder, and surgery factors.^16^ Chronic corticosteroid use has been reported to be associated with 1.2-2.4 increased risk of the dislocation after TKA.^17^^,^^18^ The explanations behind fracture and dislocation are decreased bone quality and the bone marrow density driven by corticosteroid use.^19^^,^^20^

Most previous studies were conducted in patients undergoing intra-abdominal surgeries. A meta-analysis showed that anti-TNF and corticosteroid use are associated with moderately increased 30-day postoperative infection risk in patients with IBD.^21^ Another systematic review in patients with IBD revealed that anti-TNF drugs were not shown to increase infectious complications following abdominal surgeries.^22^

With scarce data on the effect of IBD related medications on the risk of orthopedic outcomes following joint replacement surgery, multiple studies consistently showed that IBD is associated with increased risk of PJI (ranging from 1.33 to 5.44 times of increased risk) and joint revision (from 1.24 to 4.02 times).^5–7^^,^^23^^,^^24^ A meta-analysis^25^ of 8 studies discussed the risk of overall postoperative infection and joint infection in patients with IBD and arthroplasty with risk of 2.03 and 1.46, respectively, but did not investigate the risk attributed to the IBD therapies.

Our study has a number of strengths. First, our study is among the first addressing the association between therapies in IBD population and peri-PJI, joint dislocation, and wound disruption. Second, this was conducted based on a large multicenter healthcare network enhancing the generalizability of our findings. Nevertheless, several limitations must be acknowledged in this study. Medication exposure was defined as a prescription within the 3-month preceding arthroplasty; however, the dataset could not determine corticosteroid dose or duration, precise timing relative to surgery, anti-TNF dosing schedules or concentration, medication adherence, or whether therapy was held before surgery, and concomitant immunomodulator use was not accounted for in this analysis. These differences may reflect varying degrees of perioperative immunosuppression within the same exposure group. In addition, the small patient numbers in the cohort receiving newer biologics including ustekinumab, risankizumab, mirikizumab, upadacitinib, etrasimod, and ozanimod precluded our ability to analyze postoperative orthopedics outcomes on these specific medications. Moreover, residual confounding remains possible, including confounding by indication, as corticosteroid use may reflect more severe IBD and higher postoperative risk independent of medication exposure. Similarly, unmeasured differences in disease severity, inflammatory burden, prior surgery, anemia, nutritional status or healthcare utilization may remain despite propensity score matching. In addition, we were unable to perform the analysis in IBD subtypes of CD and UC due to limited outcome events. Additionally, although anti-TNF exposure was not associated with increase in PJI, confidence intervals were wide for some outcomes; therefore, a clinically meaningful increase in risk cannot be excluded. Lastly, there is no available data of indications for arthroplasty or whether the procedure was a primary or revision arthroplasty, which may influence postoperative complication risk.

While orthopedic surgeons recommend withholding anti-TNF biologic agents prior to elective TKA and THA, our data suggests that recent anti-TNF use within 3 months before surgery was not associated with a statistically significant increase in immediate PJI and dislocation. Furthermore, holding anti-TNF agents puts the patient at risk for development of anti-drug antibodies and disease flare. There has been no clear recommendation on timing of biologic agent administration in the period around elective joint replacement surgery. In another field, the American Society of Colon and Rectal surgeons recommend not to delay intra-abdominal surgical intervention in patients who are on monoclonal antibody therapy.^26^

In conclusion, when clinically feasible, minimizing corticosteroid exposure may be considered as part of perioperative optimization in patients with IBD undergoing joint replacement surgery. Anti-TNF exposure within 3 months before arthroplasty was not statistically associated with increased postoperative orthopedic complications in our study. These findings suggest that recent anti-TNF exposure may not increase postoperative risk, although larger studies are needed to confirm these findings. To optimize IBD and orthopedic care, our data support multidisciplinary collaboration between orthopedic surgery and gastroenterology to individualize perioperative management to mitigate surgical complications while avoiding IBD disease flares.

## Supplementary Material

otag119_Supplementary_Data

## Data Availability

The data used in this study were obtained from TriNetX and consist of de-identified patient information that are not publicly available due to data use restrictions. Access may be granted to accredited researchers through TriNetX subject to a data sharing agreement.
