# Supplementary material for: Association of corticosteroids and biologics use for inflammatory bowel disease with surgical outcomes following knee or hip arthroplasty
Source: Crohns Colitis 360. 2026 Nov 12;8(4):otag119. doi: 10.1093/crocol/otag119 (PMC13768107; doi:10.1093/crocol/otag119)
Supplement: Supplementary file 1 [file otag119_supplementary_data.docx]

# Supplementary Table 1: CPT and ICD-10-PCS Codes

| Code | Type | Description |
| --- | --- | --- |
| 1005073 | CPT | Arthroplasty, knee, condyle and plateau |
| 27130 | CPT | Arthroplasty, acetabular and proximal femoral prosthetic replacement (total hip arthroplasty), with or without autograft or allograft |
| 27487 | CPT | Revision of total knee arthroplasty, with or without allograft; femoral and entire tibial component |
| 27125 | CPT | Hemiarthroplasty, hip, partial (e.g., femoral stem prosthesis, bipolar arthroplasty) |
| 1004910 | CPT | Revision of total hip arthroplasty |
| 27486 | CPT | Revision of total knee arthroplasty, with or without allograft; 1 component |
| 0SWJ | ICD-10-PCS | Lower Joints / Revision / Knee Joint, Tibial Surface, Left |
| 0SRR | ICD-10-PCS | Lower Joints / Replacement / Hip Joint, Femoral Surface, Right |
| 0SRB | ICD-10-PCS | Lower Joints / Replacement / Hip Joint, Left |
| 0SRA | ICD-10-PCS | Lower Joints / Replacement / Hip Joint, Acetabular Surface, Right |
| 0SRE | ICD-10-PCS | Lower Joints / Replacement / Hip Joint, Acetabular Surface, Left |
| 0SR9 | ICD-10-PCS | Lower Joints / Replacement / Hip Joint, Right |
| 0SRC | ICD-10-PCS | Lower Joints / Replacement / Knee Joint, Right |
| 0SRD | ICD-10-PCS | Lower Joints / Replacement / Knee Joint, Left |
| 0SRL | ICD-10-PCS | Lower Joints / Replacement / Hip Joint, Femoral Surface, Left |
| 0SRV | ICD-10-PCS | Lower Joints / Replacement / Knee Joint, Tibial Surface, Right |
| 0SRT | ICD-10-PCS | Lower Joints / Replacement / Knee Joint, Femoral Surface, Right |
| 0SRU | ICD-10-PCS | Lower Joints / Replacement / Knee Joint, Femoral Surface, Left |
| 0SWC | ICD-10-PCS | Lower Joints / Revision / Knee Joint, Right |
| 0SRW | ICD-10-PCS | Lower Joints / Replacement / Knee Joint, Tibial Surface, Left |
| 0SWA | ICD-10-PCS | Lower Joints / Revision / Hip Joint, Acetabular Surface, Right |
| 0SWB | ICD-10-PCS | Lower Joints / Revision / Hip Joint, Left |
| 0SWD | ICD-10-PCS | Lower Joints / Revision / Knee Joint, Left |
| 0SW8 | ICD-10-PCS | Lower Joints / Revision / Hip Joint, Acetabular Surface, Left |
| 0SWS | ICD-10-PCS | Lower Joints / Revision / Hip Joint, Femoral Surface, Left |
| 0SWR | ICD-10-PCS | Lower Joints / Revision / Hip Joint, Femoral Surface, Right |
| 0SWT | ICD-10-PCS | Lower Joints / Revision / Knee Joint, Femoral Surface, Right |
| 0SWU | ICD-10-PCS | Lower Joints / Revision / Knee Joint, Femoral Surface, Left |
| 0SWV | ICD-10-PCS | Lower Joints / Revision / Knee Joint, Tibial Surface, Right |
| 0SW9 | ICD-10-PCS | Lower Joints / Revision / Hip Joint, Right |
